# Impact of Combination Therapy With Caffeine and Naringin on Insulin Resistance and Renal Function in a Nonalcoholic Fatty Liver Disease Rat Model

**DOI:** 10.1155/bmri/9927792

**Published:** 2026-09-17

**Authors:** Mehrasa Azizollahi, Maedeh Ghasemi, Zahra Nasehi, Mahdi Amani, Fouzieh Zadhoush

**Affiliations:** ^1^ Department of Clinical Biochemistry, School of Pharmacy and Pharmaceutical Sciences, Isfahan University of Medical Sciences, Isfahan, Iran, mui.ac.ir; ^2^ Department of Physiology, School of Medicine, Isfahan University of Medical Sciences, Isfahan, Iran, mui.ac.ir; ^3^ Pharmaceutical Sciences Research Center, School of Pharmacy and Pharmaceutical Sciences, Isfahan University of Medical Sciences, Isfahan, Iran, mui.ac.ir; ^4^ Department of Clinical Pharmacy, School of Pharmacy and Pharmaceutical Sciences, Isfahan University of Medical Sciences, Isfahan, Iran, mui.ac.ir

**Keywords:** caffeine, chronic kidney disease, naringin, nonalcoholic fatty liver disease

## Abstract

**Introduction:**

Chronic kidney disease (CKD) is a major global health concern, and evidence indicates that non‐alcoholic fatty liver disease (NAFLD) independently increases its risk and progression. This connection involves shared pathways such as chronic inflammation, oxidative stress, and insulin resistance, which can worsen renal injury. Caffeine (CAF) and naringin (NAR), with their antioxidant and anti‐inflammatory properties, may provide protective effects on both NAFLD and CKD. This study evaluated the impact of combined CAF and NAR treatment on insulin resistance and renal function in a high‐fat diet (HFD) induced NAFLD rat model.

**Materials and Methods:**

Thirty‐five male Wistar rats were randomly assigned to five groups (control, HFD, HFD + CAF, HFD + NAR, HFD + CAF + NAR; *n* = 7 per group). The HFD groups were fed a HFD (51% calories from fat) for 16 weeks, and from Week 10, received oral treatments with CAF (50 mg/kg/day), NAR (12.5 mg/kg/day), or their combination for 6 weeks. Key outcomes, including insulin resistance (HOMA‐IR), renal function (BUN, creatinine, and eGFR), and systemic inflammation (hs‐CRP) were evaluated at the study′s conclusion.

**Findings:**

HFD significantly increased glucose, HOMA‐IR, BUN, and hs‐CRP (*p* < 0.0001), as well as insulin and creatinine levels (*p* < 0.001), whereas eGFR decreased (*p* < 0.01) compared with controls. Treatment with CAF or NAR partially improved these parameters. The combination therapy produced the most pronounced effects, significantly lowering HOMA‐IR and creatinine (*p* < 0.0001), BUN and glucose (*p* < 0.001), insulin (p <0.01), and hs‐CRP levels (p <0.05), while increasing eGFR (*p* < 0.01).

**Conclusion:**

The CAF–NAR combination demonstrated superior effects compared with monotherapies in the NAFLD rat model, improving insulin resistance, enhancing renal function, and reducing systemic inflammation, thereby highlighting its potential as a promising approach for managing NAFLD‐related metabolic and renal complications.

## 1. Introduction

Chronic kidney disease (CKD) represents a substantial global health concern, contributing to elevated morbidity, mortality, and healthcare expenses. Identifying high‐risk populations and implementing early intervention are essential strategies to mitigate its progression and reduce negative outcomes. Metabolic dysfunction‐associated steatotic liver disease (MASLD), previously known as non‐alcoholic fatty liver disease (NAFLD), is now recognized as an important, yet often overlooked, risk factor for CKD [1, 2].

NAFLD, the most prevalent chronic liver disease globally, ranges from steatosis to non‐alcoholic steatohepatitis (NASH) and advanced fibrosis.

Insulin resistance is widely recognized as one of the major pathogenic mechanisms involved in the development and progression of NAFLD and is closely associated with obesity, Type 2 diabetes mellitus, dyslipidemia, and metabolic syndrome, all of which contribute to the increasing prevalence and severity of the disease [3]. By disrupting glucose and lipid metabolism, insulin resistance promotes hepatic triglyceride accumulation and steatosis, whereas persistent metabolic dysfunction further exacerbates oxidative stress and chronic inflammation, thereby accelerating liver injury and disease progression [3, 4].

Consequently, improving insulin sensitivity has emerged as an important therapeutic strategy for attenuating the progression of NAFLD [2, 5]. Increasing evidence also indicates that NAFLD is a multisystem disease with extrahepatic effects, notably renal injury. Major studies confirm that NAFLD elevates CKD risk, independent of diabetes, hypertension, and obesity, with NASH and advanced fibrosis increasing this risk further [5, 6].

NAFLD and CKD are mechanistically linked through several overlapping pathways, including altered activation of ACE‐2, impaired antioxidant defenses, and dysregulation of nutrient sensors such as sirtuin‐1. NAFLD can affect renal function through lipoprotein dysmetabolism and altered hepatokine secretion, whereas CKD can worsen NAFLD via intestinal barrier dysfunction and uremic toxin accumulation. Endothelial dysfunction and changes in adiponectin levels also appear to play a role. These connections suggest potential therapeutic targets for both diseases. Early identification and management of CKD risk in NAFLD patients are crucial, as renal impairment worsens prognosis and complicates advanced liver disease treatment. Targeting shared pathways like oxidative stress, inflammation, and metabolic dysfunction may benefit both liver and kidney health [7–10]. Since there is currently no approved pharmacological therapy for NAFLD, improving insulin sensitivity through dietary and nutritional interventions has emerged as an important therapeutic strategy for attenuating disease progression and improving metabolic outcomes [3, 11].

In this context, increasing evidence suggests that natural bioactive compounds may provide complementary therapeutic benefits in the management of NAFLD by modulating oxidative stress, inflammation, and insulin resistance. Among these compounds, caffeine (CAF) and naringin (NAR) have attracted considerable attention because of their antioxidant, anti‐inflammatory, and metabolic regulatory properties, making them promising candidates for the management of NAFLD and its metabolic complications [12, 13].

CAF, a methylxanthine in coffee and tea, exhibits antioxidant, anti‐inflammatory, and metabolic effects. Studies suggest it improves insulin sensitivity and reduces fibrosis in animal models. Its metabolic benefits, attributed to adenosine receptor antagonism, increased catecholamine concentrations, and phosphodiesterase inhibition, enhance exercise performance, thermogenesis, and lipolysis. However, the full impact of CAF on peripheral tissues and systemic metabolism requires further research [14–18].

NAR, a citrus flavonoid, exhibits nephroprotective potential in metabolic disorders. Studies indicate it alleviates oxidative stress and inflammation in diabetic kidney disease and hypercholesterolemia‐induced renal dysfunction [19, 20]. Its antioxidant mechanisms involve reducing reactive oxygen species, boosting antioxidant enzyme activity, and inhibiting lipid peroxidation [21, 22]. NAR also improved kidney function and preserved renal histology in cadmium‐induced toxicity [22]. Furthermore, it ameliorated altered purinergic signaling in the kidneys and platelets of hypercholesterolemic rats by inhibiting ROS and NF‐*κ*B pathways [20]. These findings suggest NAR′s antioxidant, anti‐inflammatory, and metal‐chelating properties contribute to renoprotection in diverse metabolic conditions, suggesting its therapeutic potential for kidney disorders. However, the combined impact of CAF and antioxidant therapy on CKD progression in NAFLD remains unclear.

Given the central roles of insulin resistance and chronic inflammation in linking NAFLD to CKD, this study is aimed at investigating the therapeutic efficacy of combined CAF–NAR treatment in a high‐fat diet (HFD)‐induced male rat model of NAFLD. We evaluated key metabolic, renal, and inflammatory markers, including HOMA‐IR, blood urea nitrogen (BUN), creatinine, estimated glomerular filtration rate (eGFR), and high‐sensitivity C‐reactive protein hs‐CRP, to provide a comprehensive assessment of treatment effects.

## 2. Materials and Methods

### 2.1. Animals

Thirty‐five male Wistar rats (190–230 g) were obtained from the animal house of the School of Pharmacy, Isfahan University of Medical Sciences. They were housed in standard polycarbonate cages (four rats per cage) under controlled conditions: 22 ± 1°C, 55% humidity, and a 12:12‐h light‐dark cycle. The animals used in the present study were maintained in accordance with the principles and guidelines of the Animal Ethics Committee of Isfahan University of Medical Sciences (approval ID: IR.MUI.AEC.1401.028).

### 2.2. Experimental Design

After 7 days of acclimatization, the rats were randomly assigned to two groups: a control group (C, *n* = 7), which received a standard laboratory chow diet containing 17% protein, 4% fat, and 65.05% carbohydrates, and a HFD group (HFD, *n* = 28), which was fed a HFD consisting of 24% protein, 26% carbohydrates, and 35% fat, with 60% of the total caloric content derived from fat, for 16 weeks. At Week 10, HFD rats were randomly divided into 4 groups (*n* = 7/group) and treated as follows for the last 6 weeks of the experimental period [23]:

1. HFD: Rats received an equivalent volume of normal saline by oral gavage.
2. HFD + CAF: Rats received CAF (50 mg/kg/day), dissolved in normal saline, by oral gavage [24].
3. HFD + NAR: Rats received NAR (12.5 mg/kg/day), dissolved in normal saline, by oral gavage [25].
4. HFD + CAF + NAR : Rats received a combination of CAF (50 mg/kg/day) and NAR (12.5 mg/kg/day), dissolved in normal saline, by oral gavage.

All drug doses were adjusted weekly according to individual body weight measurements. Following the 16‐week experimental period, all rats were fasted overnight prior to sacrifice and sample collection. Euthanasia was performed by CO_2_ asphyxiation in a controlled chamber, and blood samples were collected via abdominal aortic puncture. Serum was separated by centrifugation (3500 rpm for 10 min) within 30 min of collection and stored at −20°C until biochemical analysis.

Successful induction of NAFLD in this experimental model was confirmed previously through histopathological evaluation of hepatic steatosis, inflammation, and ballooning degeneration in the same experimental animals [23]. Furthermore, serum alanine aminotransferase (ALT) and aspartate aminotransferase (AST) levels obtained from these animals have recently been reported in a separate publication [26], further confirming the successful establishment of the NAFLD model.

### 2.3. Serum Insulin Assessments and Homeostatic Model Assessment of Insulin Resistance (HOMA‐IR) Calculation

Fasting blood glucose concentrations were quantified using a Glucose 500 mL enzymatic assay kit (Zist Shimi, Tehran, Iran). Serum insulin levels were determined using a commercially available Ultrasensitive Rat Insulin ELISA Kit (Crystal Chem Inc., Downers Grove, Illinois, United States; Catalog No. 90060). The homeostasis model assessment index of insulin resistance (HOMA‐IR) was calculated using the following formula [27]:

HOMA−IR=Fasting insulin μIU/ml×fasting glucose mmol/l 22.5.

### 2.4. BUN and Creatinine Assessment

Serum urea and creatinine concentrations were quantified using commercial reagent kits (Zist Shimi, Tehran, Iran). BUN levels were calculated using the standard conversion formula [28]:

BUN mg/dL=Urea mg/dL/2.14.

### 2.5. Measurement of eGFR

eGFR (*μ*L/min/100 g body weight) was assessed as an indicator of kidney function based on serum urea (mmol/L), serum creatinine concentration (*μ*mol/L), and body weight (g) using the following equation proposed by Besseling et al. eGFR was calculated using this rat‐specific equation, which was developed and externally validated against inulin clearance, the gold‐standard method for glomerular filtration rate measurement in rats. In the present study, eGFR was used as a comparative indicator of renal function among the experimental groups rather than as a direct measurement of GFR [29]. In this formula, *W* denotes weight, *C* represents creatinine, and *U* refers to urea.

Plasma creatinine<52880 μmol/L:eGFR= x W0.695C−0.660×U−0.391×,

Plasma creatinine≥525862 μmol/L:eGFR= x W0.695C−1.150×U−0.391×.

### 2.6. Measurement of hs‐CRP

hs‐CRP levels were quantified using a latex‐enhanced immunoturbidimetric assay (Zist Shimi, Tehran, Iran) based on enzyme immunoassay principles.

### 2.7. Statistical Analysis

Prior to statistical analysis, the normality of data distribution was assessed using the Shapiro–Wilk test, and homogeneity of variances was evaluated using Levene′s test. Data are expressed as mean ± standard error of the mean (SEM). For multiple comparisons, one‐way ANOVA followed by Tukey′s post‐hoc test was employed for parametric data, whereas the Kruskal–Wallis test followed by Dunn′s post‐hoc test was used for nonparametric analyses. All statistical analyses were performed using SPSS software (Version 26.0, IBM Corp., Armonk, New York, United States), with a *p* − value < 0.05 considered statistically significant.

## 3. Results

### 3.1. Serum Glucose, Insulin, and HOMA‐IR Levels in Study Groups

**Figure 1:**
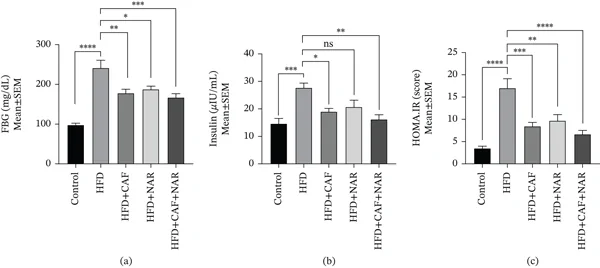
(a) Serum glucose, (b) insulin, and (c) HOMA‐IR levels in control, HFD, HFD + CAF, HFD + NAR, and HFD + CAF + NAR groups. Data represent mean ± SEM. Significance:  ^∗^ *p* < 0.05,  ^∗∗^ *p* < 0.01,  ^∗∗∗^ *p* < 0.001,  ^∗∗∗∗^ *p* < 0.0001, not significant (ns). HFD: high‐fat diet group, HFD + CAF: high‐fat diet group with caffeine (50 mg/kg/day) treatment, HFD + NAR: high‐fat diet group with naringin (12.5 mg/kg/day) treatment, HFD + CAF + NAR: high‐fat diet group with combined caffeine and naringin treatment.

Figure 1 illustrates the effects of CAF and NAR on fasting serum glucose, insulin, and HOMA‐IR levels in control and experimental rat groups. Compared with the normal control group, HFD rats exhibited significantly elevated glucose (*p* < 0.0001) and insulin (*p* < 0.001) levels. Treatment with CAF, NAR, or their combination significantly reduced serum glucose levels in all intervention groups relative to HFD controls (*p* < 0.05). However, only CAF treatment produced a significant decrease in insulin levels (*p* < 0.05), whereas NAR alone showed no statistically significant effect (ns).

However, the combination therapy of CAF and NAR demonstrated the most significant reduction in both serum glucose (*p* < 0.001) and insulin levels (*p* < 0.01) (Figure 1a,b).

The HOMA‐IR index was significantly elevated in the HFD group compared with controls (*p* < 0.0001). Both CAF (*p* < 0.001) and NAR (*p* < 0.01) treatments significantly reduced HOMA‐IR values, with the most pronounced decrease observed in the combination therapy group (*p* < 0.0001) (Figure 1c).

### 3.2. Serum Renal Function Markers in Study Groups

**Figure 2:**
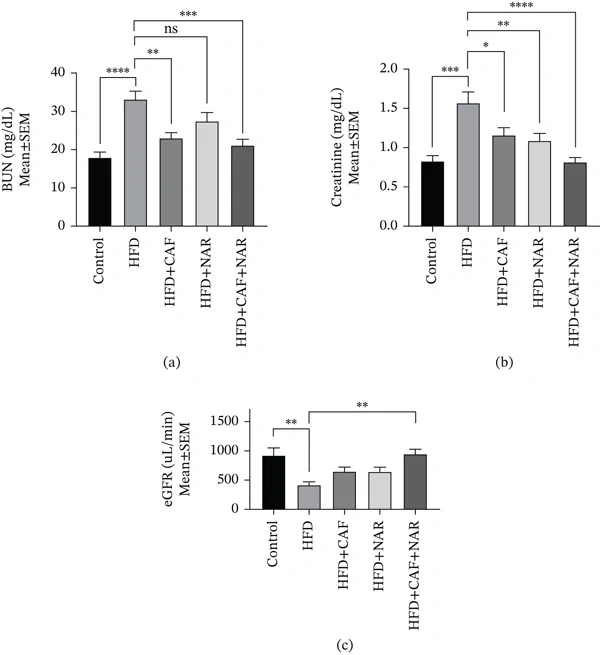
Serum biomarkers of renal function in experimental groups: (a) BUN, (b) creatinine, and (c) eGFR levels in control, HFD, HFD + CAF, HFD + NAR, and HFD + CAF + NAR groups. Data represent mean ± SEM. Significance:  ^∗^ *p* < 0.05,  ^∗∗^ *p* < 0.01,  ^∗∗∗^ *p* < 0.001,  ^∗∗∗∗^ *p* < 0.0001 versus HFD group; not significant (ns). HFD: high‐fat diet group, HFD + CAF: high‐fat diet group with caffeine (50 mg/kg/day) treatment, HFD + NAR: high‐fat diet group with naringin (12.5 mg/kg/day) treatment, HFD + CAF + NAR: high‐fat diet group with combined caffeine and naringin treatment.

Figure 2 illustrates serum levels of BUN, creatinine and eGFR across experimental groups. The HFD group showed significantly elevated BUN (*p* < 0.0001) and creatinine (*p* < 0.001) versus controls. CAF treatment significantly reduced both BUN (*p* < 0.01) and creatinine (*p* < 0.05) levels compared with the HFD group, whereas NAR treatment only lowered creatinine levels (*p* < 0.01). The CAF–NAR combination produced the most pronounced reductions in both BUN (*p* < 0.001) and creatinine (*p* < 0.0001) (Figures 2a,b).

eGFR was significantly lower in the HFD group compared with controls (*p* < 0.01). Although neither CAF nor NAR monotherapy significantly affected eGFR, the combination therapy produced a significant improvement in renal function versus HFD controls (*p* < 0.01) (Figure 2c).

### 3.3. Serum hs‐CRP Levels in Study Groups

**Figure 3:**
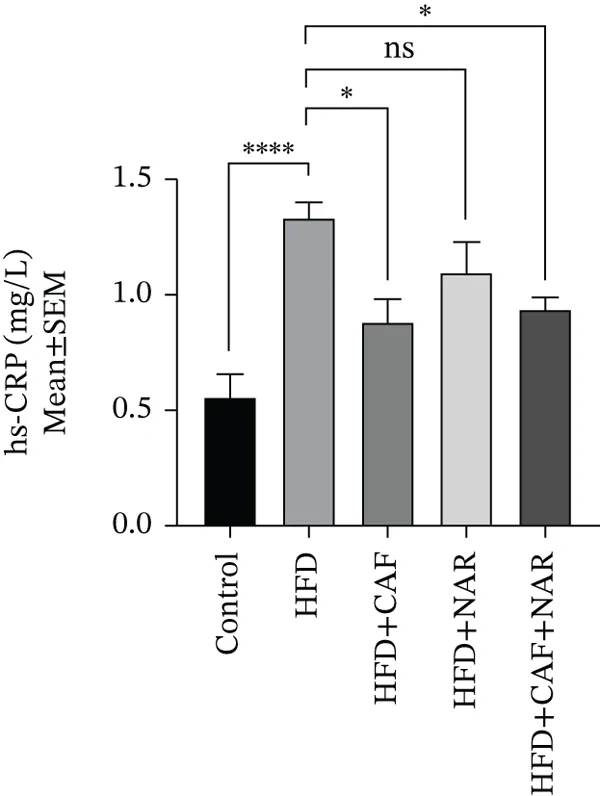
Serum hs‐CRP levels in experimental groups: control, HFD, HFD + CAF, HFD + NAR, and HFD + CAF + NAR. Data represent mean ± SEM. Significance:  ^∗^ *p* < 0.05,  ^∗∗∗∗^ *p* < 0.0001 versus HFD group. HFD: high‐fat diet group, HFD + CAF: high‐fat diet group with caffeine (50 mg/kg/day) treatment, HFD + NAR: high‐fat diet group with naringin (12.5 mg/kg/day) treatment, HFD + CAF + NAR: high‐fat diet group with combined caffeine and naringin treatment.

Serum hs‐CRP levels were markedly elevated in the HFD group relative to controls (*p* < 0.0001). CAF monotherapy significantly attenuated hs‐CRP levels after 6 weeks (*p* < 0.05), whereas NAR alone showed no significant effect (ns). The CAF–NAR combination similarly reduced hs‐CRP concentrations (*p* < 0.05) versus HFD (Figure 3).

## 4. Discussion

Accumulating evidence demonstrates a significantly increased prevalence of CKD in patients with NAFLD. This association stems from shared pathophysiological mechanisms, including insulin resistance, oxidative stress, renin‐angiotensin‐aldosterone system (RAAS) activation, and chronic inflammation mediated by proinflammatory cytokines. These findings underscore the clinical imperative to address NAFLD′s extrahepatic complications, particularly renal manifestations, in therapeutic strategies [9].

This study investigated the therapeutic effects of CAF, NAR, and their combination on metabolic and renal parameters in an HFD‐induced NAFLD rat model. Our findings indicate that the CAF–NAR combination provides superior therapeutic efficacy compared with either agent alone. The enhanced therapeutic effects of combined CAF and NAR highlight their promise as a therapeutic approach for managing the metabolic and renal complications associated with NAFLD. Consistent with prior reports [30, 31], 10 weeks of HFD administration induced significant metabolic dysregulation, evidenced by elevated fasting glucose (*p* < 0.0001), hyperinsulinemia (*p* < 0.001), and increased HOMA‐IR (*p* < 0.0001) relative to normal controls. These findings confirm the successful establishment of both systemic insulin resistance and impaired glucose homeostasis, hallmark features of NAFLD pathogenesis, in this experimental model.

Extensive mechanistic studies have established that chronic HFD consumption impairs insulin receptor substrate‐1 (IRS‐1) phosphorylation at tyrosine residues (Tyr612) while promoting inhibitory serine phosphorylation (Ser307) and subsequent Akt activation, effectively uncoupling insulin receptor signaling from its metabolic effector pathways [30, 32, 33].

CAF monotherapy significantly reduced serum glucose, insulin, and HOMA‐IR levels, consistent with previous reports demonstrating CAF′s ability to improve glucose tolerance, enhance insulin sensitivity, and ameliorate metabolic dysfunction in preclinical models [34, 35].

At the mechanistic level, CAF′s anti‐inflammatory effects—including suppression of proinflammatory cytokines (e.g., TNF‐*α* and IL‐6)—may enhance insulin signaling pathways [36, 37]. Furthermore, CAF modulates IRS‐1 phosphorylation, thereby potentiating insulin receptor activation and downstream signal transduction [38].

NAR treatment reduced blood glucose levels and improved insulin resistance without significantly affecting insulin concentrations.

These effects may be mediated, at least in part, through insulin‐independent mechanisms involving AMP‐activated protein kinase (AMPK), as suggested by previous experimental studies. AMPK activation has been reported to enhance hepatocyte GLUT2 membrane translocation and suppress hepatic gluconeogenesis, thereby promoting glucose uptake independently of insulin secretion [39–41]. However, AMPK signaling was not directly evaluated in the present study. Consistent with previous findings, NAR administration upregulates GLUT4 expression in skeletal muscle tissue, enhancing glucose metabolism in HFD‐induced obese rodent models [42]. Furthermore, NAR attenuates oxidative stress through modulation of the PI3K/Akt pathway under pro‐oxidative conditions. This mechanism contributes to enhanced insulin sensitivity via dual effects: reduction of inflammatory mediators and preservation of insulin receptor functionality [43, 44].

The CAF–NAR combination elicited more pronounced metabolic improvements than either compound administered alone. Given the complementary biological activities of CAF and NAR, coordinated regulation of AMPK, Nrf2, and NF‐*κ*B signaling represents one possible explanation for the enhanced therapeutic response. Nevertheless, the present findings do not allow definitive conclusions regarding the involvement of these pathways, and dedicated molecular studies will be required to clarify their contribution [18, 45].

Renal function assessments demonstrated significantly elevated BUN and creatinine levels accompanied by reduced eGFR in HFD‐fed animals, consistent with established associations between NAFLD and CKD progression. These findings suggest NAFLD may promote renal dysfunction through direct mechanisms, including lipotoxicity, systemic inflammation, and oxidative stress, independent of conventional cardiovascular risk factors [46, 47].

CAF administration significantly reduced serum BUN and creatinine levels. These findings are consistent with the work of Yusni et al., who demonstrated that green coffee extract (400 mg/kg) effectively lowered both BUN and creatinine in HFD‐induced obese rats [48]. Consistent with these findings, Suzuki et al. demonstrated that CAF administration, both as monotherapy and in combination with exercise, ameliorated diabetic nephropathy, as indicated by increased creatinine clearance and enhanced urinary sodium excretion, reduced proteinuria, and improved renal histology [49]. Song et al. further established that CAF‐mediated adenosine A1 receptor antagonism reduces proximal tubular sodium reabsorption, promoting natriuresis and diuresis. This mechanism lowers glomerular pressure and tubular workload, thereby attenuating hyperfiltration‐induced renal damage while improving BUN and creatinine levels [50].

NAR monotherapy significantly reduced serum creatinine levels, consistent with Zhang et al.′s report of NAR (20–80 mg/kg) improving renal function in diabetic nephropathy models through reduction of serum creatinine and urinary protein and normalization of glucose levels with in vitro studies demonstrating NAR′s capacity to attenuate ROS generation through NOX4 inhibition and suppress podocyte apoptosis via reduced cleaved caspase‐3 expression. These findings support NAR′s nephroprotective effects through dual antioxidant and anti‐apoptotic mechanisms [51].

Whereas CAF and NAR monotherapies demonstrated limited effects on eGFR, the combination therapy significantly improved renal function, as evidenced by reduced BUN and creatinine levels and enhanced eGFR.

Systemic inflammation, a key pathological feature of NAFLD, was quantified through serum hs‐CRP measurements, revealing significantly elevated levels in HFD‐fed animals relative to controls [52]. Both CAF monotherapy and combination treatment significantly attenuated hs‐CRP levels, demonstrating anti‐inflammatory efficacy. These effects align with established mechanisms whereby CAF inhibits NLRP3 inflammasome activation, modulates TLR4/MAPK/NF‐*κ*B signaling, and enhances Nrf2‐mediated antioxidant pathways [53]. However, the present study was not designed to determine which of these pathways contributed to the observed effects.

Although naringenin has demonstrated anti‐inflammatory effects through NLRP3/NF‐*κ*B pathway suppression in NAFLD models [54], our study found that NAR monotherapy did not significantly reduce hs‐CRP levels. This discrepancy may reflect differences in dosage requirements, treatment duration, and bioavailability between NAR and its aglycone form.

This study has several limitations that should be acknowledged. First, only a single dose of CAF and NAR was evaluated; therefore, potential dose‐dependent effects could not be determined. Second, the intervention period was limited to 6 weeks, precluding the assessment of the long‐term efficacy and safety of the combined treatment. Third, although renal function was assessed using serum creatinine, BUN, and a validated rat‐specific eGFR equation, direct renal function assessments, including creatinine clearance, urinary protein or albumin excretion, and renal histopathological evaluation, were not performed. Therefore, the renal findings should be interpreted in light of these limitations. Future studies incorporating multiple dose regimens, longer treatment periods, and comprehensive renal function assessments are warranted to further validate the therapeutic potential of the combined CAF–NAR therapy.

In conclusion, the combined administration of CAF and NAR demonstrated significant therapeutic efficacy in a HFD‐induced NAFLD rodent model, exhibiting superior effects compared with individual treatments. This combination therapy effectively ameliorated insulin resistance, improved renal function markers, and attenuated systemic inflammation, suggesting its potential to mitigate NAFLD‐associated metabolic and renal complications.

The observed multitarget benefits highlight the promising therapeutic potential of the combined CAF–NAR treatment. However, although preclinical results are encouraging, further investigation is required to validate these findings in human clinical trials and elucidate the precise molecular mechanisms underlying their combined therapeutic effects. Future studies should focus on optimizing dosage regimens, evaluating long‐term safety profiles, and exploring translational applications for NAFLD management.

## Funding

This study was funded by the Isfahan University of Medical Sciences (10.13039/501100003970; 3402416).

## Conflicts of Interest

The authors declare no conflicts of interest.

## Data Availability

Data are available on request.
